# AMINO ACIDS PREDICT COGNITION BEYOND CLINICAL METABOLIC MARKERS: A MACHINE LEARNING APPROACH

**DOI:** 10.1093/geroni/igz038.3456

**Published:** 2019-11-08

**Authors:** Jessie Alwerdt, Yuan Tian, Andrew D Patterson, Martin Sliwinski

**Affiliations:** 1 Penn State University, Center for Healthy Aging, University Park, Pennsylvania, United States; 2 Penn State University, Huck Institutes of the Life Sciences, University Park, Pennsylvania, United States; 3 Penn State University, The Department of Veterinary and Biomedical Sciences, University Park, Pennsylvania, United States

## Abstract

Prior work has suggested that metabolic disorders increase the risk for cognitive decline. Further, studies have identified amino acids (AAs) as potential biomarkers for dementia and diabetes. This study examines AAs and metabolic clinical markers (MCM) as predictors of cognition (Processing Speed (SOP), Working Memory (WM), Fluid (Gf) and Crystallized Intelligence (Gc)). The sample included 241 middle-aged adults from Bronx, NY. Predictors included age, gender, education, ethnicity, smoking, having diabetes, glucose, insulin, triglycerides, diastolic, and systolic blood pressure (BP), and cholesterol. AAs and associated derivatives were obtained from serum using NMR-based metabolomics. Analyses were conducted for each cognitive domain using repeated cross-validation random forests and lasso regressions. Overall, all models had acceptable cross-validation mean squared error except for WM. Several MCMs were specific to each cognitive domain, such as lower triglycerides and glucose associated with better SOP and higher systolic BP associated with better Gc while none were identified for Gf. The Gf model had the least number of AAs with lower serine associated with better FI. Two AAs, higher histidine and alanine, were associated with better SOP. Further, higher alanine, valine, isoleucine, serine, methionine, betaine, and moderate tyrosine were associated with better Gc. These results indicate that AAs were specific to each cognitive domain and ranked similar or higher in importance as several MCMs These results suggest that further investigation of AAs alongside associated MCMs is needed to assess the metabolic contribution to cognitive performance. Such research will help identify specific metabolic targets relating to cognition.

